# The Effectiveness of Virtual Reality Air‐to‐Ground Handoff Education in Realistic Operations on Learning Among Emergency Nurses in Taiwan

**DOI:** 10.1155/jonm/3797620

**Published:** 2026-03-06

**Authors:** Yu-Shan Chou, Hon-Ping Ma, Chu-Yu Huang, Chia-Jung Li, Li-Ying Chou, Su-Fen Cheng

**Affiliations:** ^1^ Department of Nursing, Shuang-Ho Hospital, Taipei Medical University, Ministry of Health and Welfare, Taipei, Taiwan, mohw.gov.tw; ^2^ Department of Allied Health Education and Digital Learning, National Taipei University of Nursing and Health Sciences, Taipei, Taiwan, ntpu.edu.tw; ^3^ Deputy Superintendent, Shuang-Ho Hospital, Taipei Medical University, Ministry of Health and Welfare, Taipei, Taiwan, mohw.gov.tw; ^4^ Graduate Institute of Injury Prevention and Control, Taipei Medical University, Taipei, Taiwan, tmu.edu.tw; ^5^ School of Nursing, Cedarville University, Cedarville, Ohio, USA, cedarville.edu; ^6^ Department of Nursing, College of Nursing, National Yang Ming Chiao Tung University, Taipei, Taiwan, nctu.edu.tw

**Keywords:** air-to-ground patient handoff, nursing education, patient handoff, psychological safety, virtual reality

## Abstract

**Aim:**

This study evaluated the effectiveness of virtual reality (VR) simulation training in enhancing emergency room (ER) nurses’ preparedness for air‐to‐ground patient handoff and psychological safety, defined as participants’ perceived confidence and comfort within the training environment.

**Background:**

Aeromedical transport is critical in regions with complex terrain or limited resources. The reception phase poses time‐sensitive and environmental challenges, and insufficient training increases the risk of handoff errors and patient harm. Existing programs focus mainly on in‐flight care, with little attention to reception. VR offers an immersive, standardized, and safe platform for reception training.

**Design:**

A quasiexperimental design using a nonrandomized, sequential cohort allocation by training date with two‐group repeated measures was employed.

**Methods:**

Seventy‐six emergency nurses from a northern Taiwan medical center were assigned by training date to a traditional drill group (*n* = 33) or a VR simulation group (*n* = 43). The “H.A.N.D.O.F.F.” curriculum, based on the 2024 CDC air‐to‐ground handoff protocol, was implemented. Outcomes included the Air‐to‐Ground Patient Handoff Preparedness Inventory and Psychological Safety Scale. Data were collected at baseline, postintervention, and 8 weeks and analyzed using generalized estimating equations.

**Results:**

Baseline score refers to participants’ initial preparedness prior to the intervention, and no significant differences were found between groups at baseline (*p* > 0.05). Postintervention, the VR group reported significantly higher preparedness and psychological safety than controls (*p* < 0.001), and these improvements were sustained at the 8‐week follow‐up (*p* < 0.001).

**Conclusions:**

This study demonstrated that VR simulation significantly improves preparedness for air‐to‐ground patient handoff, highlighting its practical value. Future applications may extend to military, police, and fire departments to foster interprofessional collaboration and enhance emergency response capabilities.

## 1. Introduction

Emergency air medical transport (EAMT) is a critical strategy to improve survival among critically ill and severely injured patients [1, 2]. A recent systematic review reported significantly reduced mortality in patients with major trauma and traumatic brain injury when transported via helicopter compared to ground ambulance [2]. Helicopter Emergency Medical Services (HEMS) provide rapid response and access to geographically challenging areas, making them essential in regions with limited infrastructure, rugged terrain, or unequal distribution of healthcare resources. For clarity, EAMT refers broadly to all forms of air medical transport (helicopter or fixed‐wing), whereas HEMS specifically denotes helicopter‐based emergency medical response. In other words, HEMS represents one operational component within the wider EAMT system [3]. Consequently, HEMS are integrated into many national emergency systems, complementing ground transport and strengthening emergency care delivery in remote and underserved settings [1, 4].

In Taiwan, mountainous landscapes and offshore islands amplify the demand for EAMT. Approximately 88% of missions involve interfacility transfers for patients requiring intensive care, most commonly with head trauma, polytrauma, or cardiovascular emergencies [5–7]. Despite its time‐sensitive benefits, EAMT presents operational challenges, including patient stabilization during flight, complex procedures within confined and unstable cabins, and critical equipment and information transfers upon landing. These processes require standardized protocols and effective interfacility coordination, underscoring the need for system‐level improvements.

EAMT is not limited to physical transfer but constitutes a continuous process of care, encompassing preflight stabilization, in‐flight emergency management, and postlanding handoff [2, 3]. Transitions of care are high‐risk events, and inadequate handoffs increase the likelihood of adverse outcomes such as airway compromise, catheter dislodgment, or monitoring disconnection [8]. Regular training, policy review, and simulation exercises are essential to promote safe and effective operations (Air and Surface Transport Nurses Association [9]).

Air‐to‐ground patient handoffs are particularly vulnerable because of four interrelated factors. First, time pressure is critical. For example, patients with acute myocardial infarction require percutaneous coronary intervention within 90 min of hospital arrival, which demands strict adherence to timelines [10]. Second, environmental constraints such as turbulence, vibration, and noise hinder procedures and communication, and ground ambulances are often preferred during cardiac arrest [11]. Third, patient instability during flight may exacerbate hemodynamic fluctuations, hypoxia, or arrhythmias [4]. Fourth, interagency coordination is frequently suboptimal; without effective communication and predefined roles, delays and errors compromise patient safety [8, 12]. These risks highlight the need for standardized handoff protocols and seamless multidisciplinary collaboration.

To address these challenges, the U.S. Centers for Disease Control and Prevention (CDC) introduced a standard operating procedure (SOP) for air‐to‐ground handoffs in 2016. This framework covers pretransport coordination, interagency communication, infection control, continuous monitoring, environmental decontamination, and structured care transfers, particularly for patients with communicable diseases. Taiwan subsequently revised its national SOPs for emergency air medical evacuation from offshore islands for emergency air evacuation in 2025, emphasizing coordination between helipads and emergency departments to improve transfer efficiency and care quality (National Aeromedical Approval Center [13]).

Although well‐established protocols exist, education and training for air medical transport remain inadequate. Current programs emphasize in‐flight clinical skills such as airway management, while communication, information exchange, and postlanding handoffs receive limited attention in simulation and evaluation [14, 15]. This imbalance leaves providers underprepared for coordination challenges, increasing the risk of clinical errors and compromised patient safety. Training is also constrained by reliance on in‐person drills, limited access to costly resources such as helicopters, and nurses’ demanding schedules and fatigue, which reduce participation [16]. Furthermore, demonstration‐based formats often prioritize observation over hands‐on practice, restricting experiential learning and situational responsiveness [17]. Consequently, current training is narrow in scope, resource‐intensive, and inaccessible, hindering nurses’ readiness for air‐to‐ground handoff responsibilities.

Beyond design and resource constraints, psychological stress in the learning environment strongly affects training effectiveness, particularly in high‐fidelity, cognitively demanding simulations [18]. Without intentional and supportive strategies, fear of mistakes diminishes engagement and learning. This reflects compromised psychological safety, defined as the confidence to share ideas and take risks without fear of criticism, a key determinant of simulation‐based learning [19, 20]. To optimize outcomes, simulations must balance challenges with support. Well‐designed simulations not only strengthen clinical skills and adaptability but also enhance emotional engagement and motivation. Educators can promote psychological safety by orienting learners to virtual reality (VR) equipment, clarifying objectives, providing clear instructional materials, explaining confidentiality of video recordings, and calibrating challenge and time pressure to ensure immersive yet manageable scenarios. Consistent positive feedback and emotional support further foster active participation and improved learning outcomes [20–22].

VR offers an innovative solution to existing training challenges. With its immersive and repeatable simulation capabilities, VR offers a multisensory, interactive learning environment where learners can safely and repeatedly practice clinical scenarios. This approach enhances scenario immersion, supports knowledge retention, and improves overall learning effectiveness [23, 24]. Compared to traditional training methods, VR simulation offers five key advantages: (1) accessibility, providing flexible, on‐demand training unconstrained by time or location; (2) standardization, ensuring consistent content delivery and assessment across learners; (3) real‐time feedback, automatically detecting errors and offering corrective guidance; (4) psychological safety, allowing learners to rehearse skills in a private, low‐pressure simulation environment without peer evaluation or performance anxiety; and (5) cost‐effectiveness, lowering long‐term costs through reusable content following initial investment [22–24]. VR simulation represents a flexible and sustainable training modality for preparing emergency nurses for air‐to‐ground patient handoffs. However, VR‐based training also has limitations. Technical issues, such as device performance, image quality, and occasional cybersickness, may affect learner comfort. Although VR can be cost‐effective long term, initial equipment and content development require investment [23]. Prior studies also note that learning benefits may diminish without reinforcement, underscoring the need for periodic refresher training [25].

Although air‐to‐ground patient handoff is an interprofessional process, this study focused on nurses because they are the primary personnel responsible for patient reception, equipment preparation, and continuity‐of‐care tasks upon landing. Their central role makes nursing preparedness critical to team performance, and establishing training effectiveness in this group provides a basis for future interprofessional expansion. Therefore, this study aimed to evaluate the effectiveness of a VR‐based air‐to‐ground patient handoff (VR‐A2G HERO) simulation program in enhancing emergency nurses’ preparedness and psychological safety during air medical patient handoffs.

## 2. Methods

### 2.1. Study Design

**FIGURE 1:**
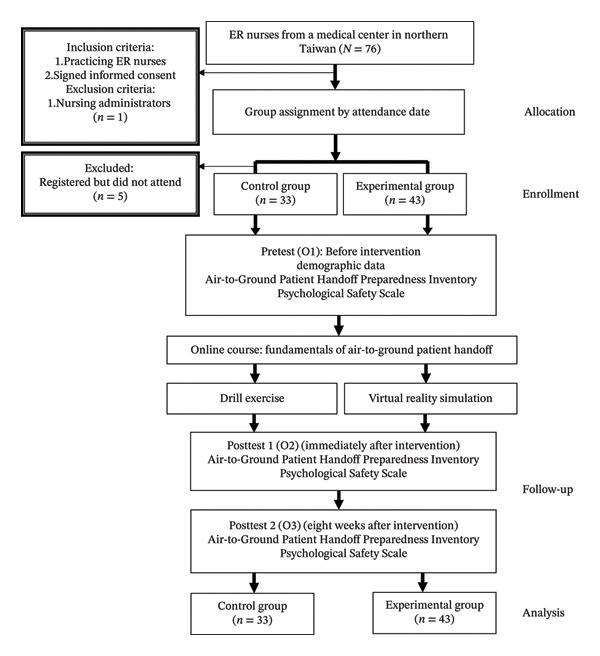
TREND study flow diagram.

This quasiexperimental study adopted a two‐group repeated measures design using convenience sampling and followed the Transparent Reporting of Evaluations with Nonrandomized Designs (TREND) reporting checklist. The experimental group received a VR‐A2G HERO: VR‐A2G HERO education in realistic operations simulation, while the control group completed a traditional drill exercise. Both groups completed a pretest before the intervention, a posttest immediately after, and a follow‐up test 8 weeks later (Figure 1).

### 2.2. Sample and Setting

Participants were emergency room (ER) nurses from a medical center in northern Taiwan. This training is part of the hospital’s mandatory continuing education program for ER nurses. The inclusion criterion was practicing ER nurses. Nurses holding administrative roles (e.g., head nurses) were excluded. To minimize cross‐group contamination, a nonrandomized sequential cohort approach was used. The control group was completed first (April–June 2025), followed by recruitment of the experimental group (June–August 2025). Sample size was calculated using G^∗^Power 3.1.9.4 with an effect size of 0.3 [25], *α* = 0.05, and power = 0.80, yielding an estimated sample of 62. To account for a 20% attrition rate, 76 participants were recruited, with 38 assigned to each group. Participants were unaware of their group assignment to either the experimental or control group. Group allocation was determined by the session enrollment date. Nurses who enrolled in the first session constituted the control group. However, five individuals were absent on the scheduled date, resulting in 33 participants. Those who enrolled in the second session formed the experimental group, which included the five nurses who missed the first session, totaling 43 participants in the experimental group. Because the intervention formats were visibly different (VR headset vs. in‐person drill), blinding of participants and instructors was not feasible. However, participants were not informed of the study hypothesis or expected differences between groups. All enrolled participants completed all three measurement time points (O1, O2, and O3), resulting in no loss to follow‐up.

### 2.3. Curriculum Planning and Design

#### 2.3.1. Needs Assessment

Before course development, an educational needs assessment questionnaire was created to identify essential training gaps related to air‐to‐ground patient handoff among ER nurses. The questionnaire was developed with reference to operational frameworks from the U.S. CDC’s SOP for air‐to‐ground patient handoff (2016) and the NAAC’s SOP for emergency air medical evacuation from offshore islands (2025). These frameworks, together with evidence from the literature review and preliminary expert interviews, informed the initial item pool. The Delphi process and exploratory factor analysis (EFA) were later used to refine item relevance and establish the questionnaire’s content and construct validity. The questionnaire covered demographic characteristics, the current status of air‐to‐ground patient handoff training, and perceptions of training importance and self‐assessed performance. Results identified key training needs in team roles and responsibilities, helicopter safety protocols, equipment preparation, and cardiac arrest procedures. These findings informed the design of the course content.

#### 2.3.2. Course Structure

The course was codeveloped by the research team. To ensure accuracy and educational rigor, the course was validated by an aeromedical and disaster medicine specialist, one critical care nursing expert, and one healthcare education specialist. The training materials, including slides and scenario scripts, were developed specifically for this study but were based on the hospital’s existing continuing education framework. All instructional content was delivered in Mandarin, which is the native language of all participants. The mnemonic H.A.N.D.O.F.F. was presented in English with an accompanying Mandarin explanation.

All participants received the fundamentals of air‐to‐ground patient handoff. Both groups first received a 10‐min foundational introduction to air‐to‐ground patient handoff procedures, included familiarization with the headset and safety protocols. The control group participated in an onsite drill exercise, while the experimental group engaged in immersive VR training. Both groups participated in postcourse feedback discussions. Both training formats were not modified based on participant feedback to maintain consistency across cohorts. The total instruction time was 60 min (Table 1).

**TABLE 1:** Comparison of treatments: control group and experimental group.

Phase	Time	Control group	Time	Experimental group
Knowledge construction	20’	Online course: fundamentals of air‐to‐ground patient handoff	20’	Online course: fundamentals of air‐to‐ground patient handoff

Air‐to‐ground handoff course content	10’	Overview of drill exercise	10’	Introduction to and trial of VR equipment
	20’	Drill exercise: Handoff site confirmed and secured Air and ground agencies aligned Notify all parties, including public health Determine transport method Outfit team with proper PPE Facilitate clean transfer Finalize documentation and debrief	15–25’	VR simulation: Handoff site confirmed and secured Air and ground agencies aligned Notify all parties, including public health Determine transport method Outfit team with proper PPE Facilitate clean transfer Finalize documentation and debrief

Supporting materials		Simulation scripts		Head‐mounted display (HMD)

Knowledge reinforcement	10’	Feedback and discussion	10’	Feedback and discussion

Total duration		60’		55–65’

#### 2.3.3. VR‐A2G HERO Course: Experimental Group

The VR‐A2G HERO course was developed based on the U.S. Centers for Disease Control and Prevention’s [26] air‐to‐ground patient handoff protocol and incorporated seven key components: (1) Handoff site confirmed and secured; (2) air and ground agencies aligned; (3) notify all parties, including public health; (4) determine transport method; (5) outfit team with proper PPE; (6) facilitate clean transfer; and (7) finalize documentation and debrief. To support retention, a mnemonic, H.A.N.D.O.F.F., was created to represent these steps.

**FIGURE 2:**
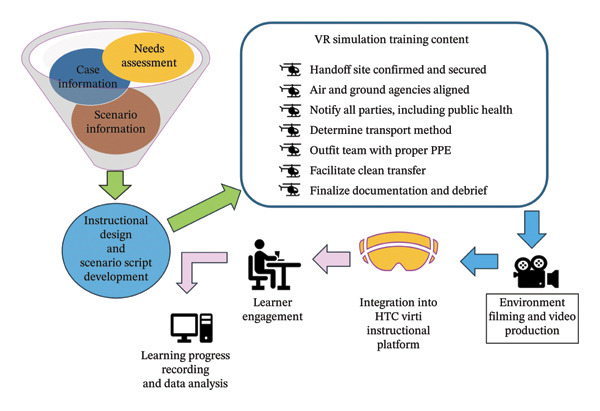
System architecture of the virtual reality simulation for air‐to‐ground patient handoff training.

To authentically represent the air‐to‐ground patient handoff scenario, this study employed a 360‐degree camera to film the entire process. The footage included receiving the emergency dispatch message from the command center, team task assignments, equipment preparation, waiting at the helipad, safety precautions when approaching the helicopter, patient handoff procedures, helicopter departure, management of cardiac arrest during transfer, and postarrival documentation and debriefing in the emergency department. The 360‐degree video was then edited and integrated into an instructional design using HTC’s Virti platform. Due to the challenges of capturing certain scenes, such as helicopter dispatch, takeoff and landing, and news footage, artificial intelligence (AI) technology was used to generate supplemental visuals. Sound effects were added to simulate the urgency and intensity of helicopter operations. Participants experienced the simulation using a economically accessible head‐mounted display (HMD), the VR Box 3rd Generation (VR glasses), which allowed first‐person immersive viewing of the 360° scenario. The device costs approximately USD $6.5, making it feasible for adoption in clinical settings. The VR design was developed by our team and took about 3 weeks to complete. The training scenario opened with a news clip depicting a mountain rescue and subsequent helicopter transfer, creating contextual immersion. Participants were then guided through the air‐to‐ground handoff procedures using task‐based prompts. The simulation incorporated interactive elements such as multiple‐choice questions, timers, and decision‐making options to enhance engagement and learning outcomes. Incorrect responses prompted a replay of relevant video segments to reinforce understanding. Upon completion, participants received feedback on their total task time and response accuracy, supporting self‐reflection and performance evaluation. The VR‐A2G HERO training session is self‐paced. Each participant completed the full scenario once under supervision. Participants were trained in supervised sessions and were allowed to raise their hand or pause the scenario at any time to ask questions during VR use. The training time ranged from 15 to 25 min depending on individual learning progress. All data were collected and stored on the instructional platform’s backend for further analysis and integration (Figure 2).

#### 2.3.4. Drill Exercise: Control Group

The control training was based on a routine educational activity implemented in our institution. The control group received a single instructor‐led session simulating the air‐to‐ground reception process, with an ER physician, security officer, and facility engineer participating. The instructor demonstrated the procedures for all participants, who could ask questions throughout. The session addressed seven core concepts: (1) handoff site confirmed and secured; (2) air and ground agencies aligned; (3) notify all parties, including public health; (4) determine transport method; (5) outfit team with proper PPE; (6) facilitate clean transfer; and (7) finalize documentation and debrief. Elements that could not be realistically simulated (patient reception, helicopter takeoff/landing, and cardiac arrest during transfer) were explained conceptually by the instructor. A postsession discussion and feedback period reinforced key concepts.

### 2.4. Pilot Testing of VR‐Based Simulation

This study invited three emergency nurses from nonparticipating sites to pilot the VR‐A2G HERO virtual reality air‐to‐ground patient handoff simulation. The pilot aimed to evaluate system functionality and interface clarity. Regular quality monitoring of the training materials was conducted to ensure data reliability and completeness throughout the study period.

### 2.5. Data Collection and Instruments

#### 2.5.1. Demographic Information

Data collected were gender, age, education level, emergency nursing experience, clinical ladder level, prior experience with air‐to‐ground patient handoff, and prior training in air‐to‐ground patient handoff reception.

#### 2.5.2. Air‐to‐Ground Patient Handoff Preparedness Inventory

The Air‐to‐Ground Patient Handoff Preparedness Inventory was adapted from the 44‐item Emergency Preparedness Information Questionnaire developed by Garbutt et al. [27]. To develop a handoff‐specific instrument, 20 items relevant to air‐to‐ground communication, coordination, and operational readiness were initially retained. Content validity was established through a two‐round Delphi review involving three experts: an aeromedical and disaster medicine specialist, one acute/critical care nursing expert with experience in air‐to‐ground patient handoff, and one healthcare education specialist. During the first Delphi round, four items were removed due to redundancy or limited relevance, and several wording modifications were made for clarity. The second round resulted in a refined 16‐item version with excellent content validity (I–content validity index [CVI] = 1.0; S‐CVI = 1.0).

The original English instrument was translated into Mandarin using a forward‐translation process conducted by an English‐language specialist, and the Mandarin version was used for data collection. Construct validity was examined using EFA. The Kaiser–Meyer–Olkin (KMO) value was 0.931, and Bartlett’s test of sphericity was significant (*p* < 0.001), indicating suitability for factor analysis. Principal component analysis with varimax rotation extracted six factors with eigenvalues greater than 1, accounting for 95.408% of the total variance. The final instrument consists of 16 items across six domains: communication and coordination, triage, command systems, isolation and protection, psychological/special needs, and reporting and resource acquisition. In this study sample, the instrument demonstrated excellent internal consistency, with a Cronbach’s *α* of 0.98. Responses were rated on a 5‐point Likert scale, where 1 indicated “strongly disagree” and 5 indicated “strongly agree.” Higher scores reflected greater preparedness for air‐to‐ground patient handoff.

#### 2.5.3. Psychological Safety Scale

Psychological safety was measured using the 14‐item Psychological Safety Scale by Park and Kim [28], which covered the following four domains: managing uncertainty, exposure to risk, lack of external support, and interpersonal risk. Responses were measured using a 5‐point Likert scale, where 1 indicated “strongly disagree” and 5 indicated “strongly agree”. Higher scores reflected a greater sense of psychological safety. The scale demonstrated strong content validity, with a CVI of 0.94, and high internal consistency, with a Cronbach’s *α* of 0.91.

### 2.6. Statistical Analysis

Data were analyzed using SPSS IBM 29.0. Descriptive statistics were used to summarize participant characteristics. Independent *t*‐tests and paired *t*‐tests were conducted for descriptive comparisons between and within groups. Standardized mean differences (Hedges’ *g*) with 95% confidence intervals were calculated post hoc based on group means, pooled standard deviations, and sample sizes to quantify between‐group effect sizes. Effect size magnitudes were interpreted according to Cohen’s criteria, with values of approximately 0.2 indicating a small effect, 0.5 a medium effect, and 0.8 or greater a large effect [29]. The primary analysis was performed using generalized estimating equations (GEEs) to examine the effects of the intervention over time. All participants completed all measurement time points, and there was no attrition. Therefore, GEE was appropriate for estimating the average intervention effect at the population level, and additional sensitivity analyses were not required. An autoregressive correlation structure (AR[1]) was specified to model within‐subject dependence across repeated measurements, and robust standard errors were applied.

### 2.7. Ethical Consideration

This study was approved by the institutional review board (IRB) of a medical center (IRB No. N202412086; approval date: February 28, 2025). The study was conducted in accordance with IRB guidelines. Before the training session, participants received a standardized briefing describing the study purpose and procedures. They were informed that two instructional formats were being implemented during the study period. All participating nurses provided written informed consent prior to enrollment.

The VR system did not include any voice or image recognition functions. No identifiable audio or visual data of participants were captured during the VR sessions. Questionnaire responses were fully anonymous and collected without any personal identifiers. All study‐related data were stored solely by the principal investigator on a password‐protected personal computer. In accordance with IRB requirements, all digital files, including those stored on local drives and institutional cloud storage (Google Drive), will be permanently deleted 3 years after study completion or termination.

## 3. Results

### 3.1. Demographic Characteristics

Baseline equivalence between the two groups was assessed using independent *t*‐tests and chi‐square tests for demographic and clinical characteristics. A total of 76 ER nurses participated in this study (control group: *n* = 33, 43.4%; experimental group: *n* = 43, 56.6%). Most participants were female (88.2%), with a mean age of 30.6 years (SD = 6.8) and a mean work experience of 89.8 months (SD = 67.3). The majority (82.9%) had no prior experience in air‐to‐ground patient handoff, and 73.7% had not received related training. Of the 26.3% who had received related training, most had participated in traditional onsite drill exercises. There were no significant differences between groups in baseline characteristics (*p* > 0.05) (Table 2).

**TABLE 2:** Demographic characteristics.

Variables	Total (*n* = 76)	CON (*n* = 33)	EXP (*n* = 43)	*t*/*χ* ^2^	*p*
	*M* ± SD/*n* (%)	*M* ± SD/*n* (%)	*M* ± SD/*n* (%)		
Gender				1.867^a^	0.172
Male	9 (11.8%)	2 (6.1%)	7 (16.3%)		
Female	67 (88.2%)	31 (93.9%)	36 (83.7%)		
Age	30.6 ± 6.75	30.2 ± 6.43	30.9 ± 7.05	−0.438^b^	0.663
20–29 y/o	35 (46.1%)	15 (45.5%)	20 (46.5%)		
30–39 y/o	27 (35.5%)	12 (36.4%)	15 (34.9%)		
> 40 y/o	14 (18.4%)	6 (18.1%)	8 (18.6%)		
Work experience (months)	89.8 ± 67.28	87.2 ± 68.61	91.8 ± 66.98	−0.294^b^	0.769
< 60 m	28 (36.8%)	13 (39.4%)	15 (34.9%)		
60–119 m	24 (31.6%)	9 (27.3%)	15 (34.9%)		
> 120 m	24 (31.6%)	11 (33.3%)	13 (30.2%)		
Prior air‐to‐ground handoff experience				0.157^a^	0.692
Yes	13 (17.1%)	5 (15.2%)	8 (18.6%)		
No	63 (82.9%)	28 (84.8%)	35 (81.4%)		
Prior training experience				1.990^a^	0.158
Yes	20 (26.3%)	6 (18.2%)	14 (32.6%)		
No	56 (73.7%)	27 (81.8%)	29 (67.4%)		

*Note:* CON = control group; EXP = experimental group.

^a^Pearson chi‐square.

^b^ *t*‐test.

### 3.2. Difference in Air‐to‐Ground Patient Handoff Preparedness

#### 3.2.1. Independent Samples *t*‐Test

No significant differences were found between groups in preparedness scores at O1 (*t* = −0.626, *p* > 0.05), indicating group homogeneity. After the intervention, the experimental group’s preparedness score increased by 37.6% from baseline to posttest, with a mean difference of 15.7 points on an 80‐point scale, corresponding to a large effect size (Hedge’s *g* = 1.30, 95% CI: 0.79–1.80). The experimental group also scored significantly higher than the control group at O2 (*t* = −5.465, *p* < 0.001). At the 8‐week follow‐up (O3), the experimental group continued to demonstrate significantly higher scores compared to the control group (*t* = −2.235, *p* < 0.05, Hedge’s *g* = 0.81, 95% CI: 0.33–1.29) (Table 3).

**TABLE 3:** Differences in air‐to‐ground patient handoff preparedness and psychological safety scores between groups before and after simulation‐based training interventions.

Variable		Total (*n* = 76)	CON (*n* = 33)	EXP (*n* = 43)	*t*(*p*)	Mean difference (CON − EXP)	Hedges’ *g*
		*M* ± SD	*M* ± SD	*M* ± SD		(95% CI)	(95% CI)
Preparedness	O1	48.6 ± 16.15	47.3 ± 16.52	49.7 ± 15.99	−0.626 (*p* > 0.05)	−2.4 (−9.82, 5.13)	N/A
	O2	61.6 ± 14.00	52.7 ± 14.35	68.4 ± 9.18	−5.465 (*p* < 0.001)	−15.7 (−21.40, −9.90)	1.30 (0.79–1.80)
	O3	58.6 ± 17.87	53.3 ± 20.51	67.7 ± 14.52	−2.235 (*p* < 0.05)	−14.4 (−17.80, −0.96)	0.81 (0.33–1.29)

Psychological safety	O1	41.6 ± 11.97	40.2 ± 10.98	42.7 ± 12.70	−0.924 (*p* > 0.05)	−2.5 (−8.08, 2.96)	N/A
	O2	46.7 ± 13.53	37.8 ± 12.86	53.6 ± 9.50	−6.161 (*p* < 0.001)	−15.8 (−20.90, −10.68)	1.41 (0.89–1.92)
	O3	47.1 ± 13.37	42.5 ± 14.25	50.7 ± 11.60	−2.770 (*p* < 0.01)	−8.2 (−14.12, −2.30)	0.63 (0.16–1.10)

*Note:* No loss to follow‐up occurred; *n* remained constant across O1, O2, and O3 for both groups. CON = control group; EXP = experimental group; O1 = baseline; O2 = posttest; O3 = 8‐week follow‐up. The maximum score for the Air‐to‐Ground Patient Handoff Preparedness Inventory was 80, and the maximum score for the Psychological Safety Scale was 70. Mean differences were calculated as control group minus the experimental group (CON − EXP); therefore, negative values indicate higher scores in the experimental group.

#### 3.2.2. GEE Analysis

**FIGURE 3:**
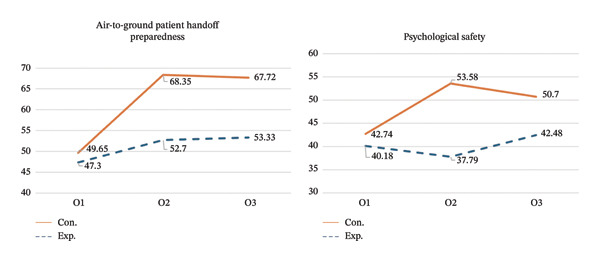
Differences in air‐to‐ground patient handoff preparedness and psychological safety scores between the two groups.

GEE was used to analyze changes in preparedness scores over time, with the control group as the reference. From O1 to O2, the experimental group demonstrated an improvement of 18.69 points, which is greater than that of the control group, a difference that was statistically significant (*p* < 0.001). Given that the preparedness scores have a maximum score of 80, this difference represents an improvement of approximately 23.4% of the total scale range with a large effect size (Hedges’ *g* = 1.30). From O1 to O3, the experimental group continued to show a statistically significantly greater improvement, exceeding the control group by 13.07 points (*p* < 0.001), corresponding to approximately 16.3% of the maximum possible score, indicating a sustained and clinically meaningful improvement over time with a large effect size (Hedges’ *g* = 0.81). These findings indicate that the intervention effects were maintained at the 8‐week follow‐up (Table 4 and Figure 3).

**TABLE 4:** Summary results of the generalized estimating equation analysis.

Variable	GEE			
	Air‐to‐ground patient handoff preparedness		Psychological safety	
	*β*	*p*	*β*	*p*
Intercept	47.30	< 0.001	40.18	< 0.001
Group (EXP vs. CON)	2.35	0.528	2.56	0.340
Time				
O3 vs. O1	6.03	0.135	2.30	0.507
O2 vs. O1	5.39	< 0.001	−2.39	0.239
Group × time				
EXP (O3 vs. O1)	13.07	< 0.001	7.95	< 0.001
EXP (O2 vs. O1)	18.69	< 0.001	10.84	< 0.001

*Note:* CON = control group; EXP = experimental group; O1 = baseline; O2 = posttest; O3 = 8‐week follow‐up.

### 3.3. Differences in Psychological Safety

#### 3.3.1. Independent Samples *t*‐Test

No significant differences were observed between groups in psychological safety scores at O1 (*t* = −0.924, *p* > 0.05), indicating group homogeneity. After the intervention, the experimental group’s psychological safety score increased by 25.4% from baseline to posttest, with a mean difference of 15.8 points on a 70‐point scale, corresponding to a very large effect size (Hedges’ *g* = 1.41, 95% CI: 0.89–1.92). The experimental group also scored significantly higher than the control group at O2 (*t* = −6.161, *p* < 0.001). At the 8‐week follow‐up (O3), the experimental group continued to show significantly higher scores than the control group (*t* = −2.770, *p* < 0.01, Hedges’ *g* = 0.63, 95% CI: 0.16–1.10) (Table 3).

At O1, the highest psychological safety dimension was dealing with uncertainty in the experimental group (3.10 ± 1.04) and interpersonal risk in the control group (3.07 ± 0.83). At O2, being unsupported showed the highest mean score in the experimental group (3.96 ± 0.86), whereas interpersonal risk remained the highest in the control group (3.00 ± 0.95). At O3, being unsupported had the highest mean score in both the experimental group (3.96 ± 0.86) and the control group (3.17 ± 1.09) (Table 5).

**TABLE 5:** Psychological safety domain‐level.

Variable	CON (*n* = 33) *M* ± SD	EXP (*n* = 43) *M* ± SD	*t(p)*	95% CIs	
				LL	UL
*O1*					
Dealing with uncertainty	3.02 ± 0.87	3.10 ± 1.04	−0.364 (*p* > 0.05)	0.366	−0.530
Being exposed	2.72 ± 0.95	3.02 ± 1.05	−1.279 (*p* > 0.05)	0.166	−0.762
Being unsupported	2.67 ± 1.00	3.09 ± 1.10	−1.713 (*p* > 0.05)	0.682	−0.905
Interpersonal risk	3.07 ± 0.83	3.00 ± 0.87	0.358 (*p* > 0.05)	0.464	−0.323

*O2*					
Dealing with uncertainty	2.64 ± 0.96	3.93 ± 0.78	−6.423 (*p* < 0.001)	−0.887	−1.685
Being exposed	2.67 ± 1.05	3.93 ± 0.83	−5.701 (*p* < 0.001)	−0.820	−1.707
Being unsupported	2.52 ± 1.08	3.96 ± 0.86	−6.515 (*p* < 0.001)	−1.003	−1.888
Interpersonal risk	3.00 ± 0.95	3.42 ± 0.90	−1.958 (*p* > 0.05)	0.007	−0.844

*O3*					
Dealing with uncertainty	2.99 ± 1.02	3.73 ± 0.90	−3.352 (*p* < 0.001)	−0.300	−1.180
Being exposed	3.04 ± 1.09	3.69 ± 0.90	−2.831 (*p* < 0.01)	−0.192	−1.104
Being unsupported	3.17 ± 1.09	3.96 ± 0.86	−6.515 (*p* < 0.001)	−1.003	−1.888
Interpersonal risk	2.95 ± 1.04	3.27 ± 0.89	−1.455 (*p* > 0.05)	0.119	−0.763

*Note:* No loss to follow‐up occurred; *n* remained constant across O1, O2, and O3 for both groups. CON = control group; EXP = experimental group; O1 = baseline; O2 = posttest; O3 = 8‐week follow‐up. Group comparisons were conducted using independent samples *t* tests (control − experimental). Negative *t* values and confidence intervals indicate higher scores in the experimental group.

#### 3.3.2. GEE Analysis

GEE was conducted to analyze changes in psychological safety scores over time, with the control group as the reference. From O1 to O2, the experimental group showed a 10.84‐point greater improvement than the control group, which was statistically significant (*p* < 0.001). Given that the psychological safety scale has a maximum score of 70, this difference represents an improvement of approximately 15.5% of the total scale range, with a large effect size (Hedges’ *g* = 1.41). From O1 to O3, the experimental group demonstrated a 7.95‐point greater improvement than the control group (*p* < 0.001), corresponding to approximately 11.4% of the maximum possible score with medium effect size (Hedges’ *g* = 0.63) (Table 4 and Figure 3).

## 4. Discussion

This study employed the “VR‐A2G HERO” as a simulation training intervention for air‐to‐ground patient handoff. Findings indicated that emergency nurses who participated in the program demonstrated significant improvements in both handoff preparedness and psychological safety compared to the control group, with sustained effects at follow‐up. It is important to note that the improvements observed in this study were based on self‐reported measures rather than direct assessments of clinical performance.

### 4.1. Air‐to‐Ground Patient Handoff Preparedness

The VR‐A2G HERO utilized 360‐degree immersive environments combined with AI technology to realistically replicate each stage of the air medical reception process. The program incorporated interactive questioning and feedback mechanisms to guide nurses through the handoff procedure. Results showed that VR simulation significantly enhanced nurses’ preparedness for patient reception and that these effects persisted up to 8 weeks postintervention (*p* < 0.001). These findings align with existing literature supporting VR as an effective tool in emergency response and clinical education [25, 30, 31].

Yang and Oh [30] designed an immersive VR‐based gamified neonatal resuscitation course grounded in the 2020 American Heart Association guidelines, reporting improvements in knowledge, clinical reasoning, motivation, and confidence among nursing students. Chang et al. [25] applied 360° VR to simulate chemical disaster patient management in the emergency department, finding enhanced preparedness and self‐efficacy among emergency nurses. Lee and Han [31] developed VR training for ventilator management in COVID‐19 patients with ARDS, demonstrating improved knowledge, reasoning, self‐efficacy, and satisfaction. A systematic review by Tamilselvan et al. [17] further noted that VR overcomes limitations of traditional simulation in terms of resources and learner participation, providing flexible, scalable, and safe training opportunities. Similar to these studies, our VR‐based emergency response training also demonstrated meaningful improvements, with participants reporting significantly enhanced preparedness for air‐to‐ground patient handoff.

Although air‐to‐ground handoffs occur infrequently in this ER, they represent high‐acuity, low‐frequency events that require accurate and coordinated responses. The VR‐A2G HERO provides a standardized and repeatable platform that allows nurses to practice these procedures despite limited real‐world exposure. In addition, the low cost and flexible scheduling of VR training support its use for periodic refreshers, thus maintaining readiness even when clinical opportunities are scarce. Consistent with our findings, Chang et al. [25] also noted that VR is well‐suited as a presimulation preparatory activity, helping learners build foundational readiness before participating in live drills.

From a feasibility and resource perspective, the development of the VR‐A2G HERO required approximately three weeks, including instructional design, 360‐degree video production, and platform setup. The program was delivered via a paid institutional VR platform (HTC Virti) and implemented using a low‐cost HMD (approximately USD 6.5), supporting scalability and accessibility. Each VR training session required approximately 15–25 min and could be completed flexibly without the need for instructor presence or coordinated scheduling. In contrast, traditional onsite drills typically require coordination among multiple healthcare personnel, dedicated instructors, and scheduled training time of at least 1 hour, resulting in higher recurring personnel and time costs for each session [32]. Once developed, the VR‐A2G HERO can be reused without additional instructional time and easily adapted for refresher training or future content updates. Although VR‐based training involves greater upfront development investment, it minimizes per‐learner costs after implementation. While a formal cost‐effectiveness analysis was not conducted in this study, these characteristics suggest that VR‐based training may offer practical advantages over traditional training approaches in terms of scalability, flexibility, and long‐term resource efficiency.

In contrast, some studies have reported mixed findings. Smith et al. [33] found no significant differences between VR and live simulation for decontamination skills, though methodological limitations such as the absence of pre–post testing reduced the strength of evidence. Chao et al. [34] similarly found that immersive 3D video training for enteral feeding improved knowledge but was not superior to traditional video, with learners citing extra time demands and physical discomfort from HMDs. Miguel‐Alonso et al. [35] highlighted the “novelty effect,” suggesting that initial enthusiasm for VR may inflate short‐term outcomes but not necessarily sustain long‐term benefits. They recommended presession orientation to mitigate this effect and improve learner familiarity.

This study addressed these limitations by using eye‐tracking headsets that enabled interaction through gaze rather than handheld controllers, thus reducing technical burdens. Pretraining orientation, immediate posttesting, and delayed assessments at 8 weeks helped minimize novelty effects and verify sustained outcomes. Approximately 30% of learners reported experiencing mild dizziness while using VR, primarily during running scenarios. No moderate or severe dizziness was observed during the study. Learners indicated that familiarizing themselves with the equipment beforehand may have helped reduce discomfort while using VR, as all symptoms were transient and did not affect course completion. The VR‐A2G HERO uniquely integrated AI‐enhanced realism, multirole interactions, multiple‐choice response mechanisms, and real‐time feedback, offering a cost‐effective yet highly immersive platform. These design features provide evidence for VR’s durable effectiveness in preparing nurses for complex handoff scenarios. Although preparedness improved, it remains uncertain whether these gains translate into enhanced clinical performance. Future studies may incorporate OSCE assessments or skill‐based performance checklists and include a longer follow‐up period to determine whether VR training not only increases confidence but also leads to improvements in practice.

### 4.2. Psychological Safety

This study also found that psychological safety declined among nurses in the traditional simulation group but significantly increased and persisted among those in the VR group (*p* < 0.001). It indicated that the experimental group has better psychological safety. Prior studies have often relied on qualitative data to examine psychological safety in simulation education [21, 36], with few using validated scales. By contrast, the present study quantitatively assessed psychological safety and demonstrated VR’s advantage in fostering safe, supportive learning environments.

The findings are consistent with prior research showing that immersive, nonjudgmental VR environments enhance psychological safety. Dale‐Tam et al. [36] reported improvements when combining 360° VR videos with online discussions and positive feedback. Kiegaldie and Shaw [32] found that VR reduced fear and stress compared to traditional simulation, promoting greater participation and repetition. Kim et al. [20] emphasized that while psychological safety is critical, it can be undermined by learner anxiety and uncertainty.

Subscale analysis in this study revealed that the control group scored lowest on “dealing with uncertainty” and “being unsupported,” while the VR group scored highest in these domains. Dealing with uncertainty reflects learners’ anxiety when facing unfamiliar equipment, time pressure, or potential errors, whereas being unsupported relates to concerns about negative feedback or limited teacher–learner interaction. The VR‐A2G HERO reduced uncertainty through presession orientation and enhanced support through immediate positive feedback, thus fostering psychological safety.

These findings echo prior work indicating that greater simulation experience [21] and responsive faculty facilitation [37] promote psychological safety. In contrast, traditional group–based simulations often limit individualized observation and timely feedback, leaving some learners anxious about performing under peer scrutiny. The VR‐A2G HERO, by enabling self‐paced, repeatable practice, overcomes manpower and space limitations of live drills and provides a scalable model for sustainable education.

### 4.3. Limitations

This study was limited to ER nurses from a single medical center, with a relatively small and homogenous sample, which may constrain the generalizability of the findings. Future studies should consider including participants from multiple hospital systems, diverse types of medical institutions, and interprofessional disciplines to enhance the external validity and broaden the applicability of the findings. The study also employed a nonrandomized, sequential cohort design, which introduces the possibility of selection or history bias, and neither participants nor instructors could be blinded due to the visibly distinct training formats. Outcomes were assessed using self‐reported measures of preparedness and psychological safety rather than objective performance evaluations. As a result, the results may reflect perceived rather than demonstrated competence. In addition, although both groups received a single training exposure, the nature of practice opportunities differed. The VR group engaged in an individualized immersive scenario, whereas the control group participated in a group‐based drill, which may have led to unequal task practice. Moreover, despite a presession orientation, novelty or expectancy effects cannot be fully excluded, particularly given the immersive nature of VR‐based learning. Finally, approximately 30% of participants reported dizziness or discomfort when using the HMD, primarily during running scenarios. Physiological tolerance to VR remains a challenge. Future studies should explore alternative display devices or phased adaptation strategies to minimize discomfort and improve the overall learning experience.

## 5. Conclusion

This study found that participation in the VR‐A2G HERO program was associated with higher preparedness for air‐to‐ground patient reception and improved psychological safety among emergency nurses, suggesting the potential value of a VR‐based, feedback‐rich simulation model for clinical education. These findings reflect associations rather than causal effects, given the nonrandomized study design. Future research should explore its application across broader healthcare teams to meet the collaborative demands of aeromedical transfer. Multisite replication is also recommended to enhance generalizability and to evaluate how different institutional contexts influence training outcomes. Educators may also adapt this approach to develop course content and learning objectives that convert rare or high‐risk scenarios into VR‐based training modules for continuing nursing education and professional development. Such innovations strengthen the link between knowledge and clinical practice and offer a scalable framework for advancing simulation‐based education in emergency and critical care settings.

## Author Contributions

This is our original, unpublished work and has not been submitted to other journals. All authors made significant contributions to this study.

## Funding

This work was supported by the Taipei Medical University‐Shuang‐Ho Hospital, Ministry of Health and Welfare (114‐ME03, 2025).

## Conflicts of Interest

The authors declare no conflicts of interest.

## Data Availability

The data that support the findings of this study are available on request from the corresponding author. The data are not publicly available due to privacy or ethical restrictions.
